# Transferable CNN-based data mining approaches for medical imaging: application to spine DXA scans for osteoporosis detection

**DOI:** 10.3389/fncom.2025.1712896

**Published:** 2025-12-30

**Authors:** Awad Bin Naeem, Onur Osman, Shtwai Alsubai, Nazife Çevik, Abdelhamid Taieb Zaidi, Amir Seyyedabbasi, Jawad Rasheed

**Affiliations:** 1 National College of Business Administration and Economics, Multan, Pakistan; 2 Department of Biomedical and Neuromotor Sciences, University of Bologna, Bologna, Italy; 3 Department of Electrical and Electronics Engineering, Istanbul Topkapi University, Istanbul, Türkiye; 4 Department of Computer Science, College of Computer Engineering and Sciences in Al-Kharj, Prince Sattam Bin Abdulaziz University, Al-Kharj, Saudi Arabia; 5 Department of Computer Engineering, Istanbul Arel University, Istanbul, Türkiye; 6 Department of Mathematics, College of Science, Qassim University, Buraydah, Saudi Arabia; 7 Department of Computer Engineering, Istinye University, Istanbul, Türkiye; 8 Department of Computer Engineering, Istanbul Sabahattin Zaim University, Istanbul, Türkiye; 9 Department of Software Engineering, Istanbul Nisantasi University, Istanbul, Türkiye; 10 Research Institute, Istanbul Medipol University, Istanbul, Türkiye; 11 Applied Science Research Center, Applied Science Private University, Amman, Jordan

**Keywords:** CNN, classification model, osteoporosis, DXA images, image processing, medical diagnosis

## Abstract

**Introduction:**

Osteoporosis is the leading cause of sudden bone fractures. This is a silent and deadly disease that can affect any part of the body, such as the spine, hips, and knee bones.

**Aim:**

To measure bone mineral density, dual-energy X-ray absorptiometry (DXA) scans help radiologists and other medical professionals identify early signs of osteoporosis in the spine.

**Methods:**

A proposed 21-layer convolutional neural network (CNN) model is implemented and validated to automatically detect osteoporosis in spine DXA images. The dataset contains 174 spine DXA images, including 114 affected by osteoporosis and the rest normal or non-fractured. To improve training, the dataset is expanded using various data augmentation techniques.

**Results:**

The classification performance of the proposed model is compared with that of four popular pre-trained models: ResNet-50, Visual Geometry Group 16 (VGG-16), VGG-19, and InceptionV3. With an F1-score of 97.16%, recall of 95.41%, classification accuracy of 97.14%, and precision of 99.04%, the proposed model consistently outperforms competing approaches.

**Conclusion:**

The proposed paradigm would therefore be very valuable to radiologists and other medical professionals. The proposed approach’s capacity to detect, monitor, and diagnose osteoporosis may reduce the risk of developing the condition.

## 1 Introduction

With advances in medicine, the average life expectancy has increased, resulting in an increasingly elderly population. Aging is the most significant and common factor in bone diseases, as bones lose their strength with age. Hip and vertebral fractures are linked with inpatient and body disorders that are major causes of bone disease. In Europe, almost 27.5 million people are reported to have osteoporosis. The most common disease causing these fractures is osteoporosis, characterized by a loss of bone mineral density. A patient’s bone mineral density (BMD) value can help a physician minimize the risk of these fractures. In this era, with the increasing population, the cases of osteoporosis are increasing day by day, especially in older adults, because they have low BMD. In 2010, the number of lives lost due to fractures in Europe was 1,180,000. Therefore, it is crucial to minimize these fractures. To determine bone mineral density (BMD), Dual-energy X-ray absorptiometry (DXA) is used (Maki et al., 2024). This determination underscores the need for a high-resolution dual-energy X-ray absorptiometry (DXA) bone densitometer to evaluate bone mineral density, bone mineral content, and body composition (Lai et al., 2024).

**Figure 1:**
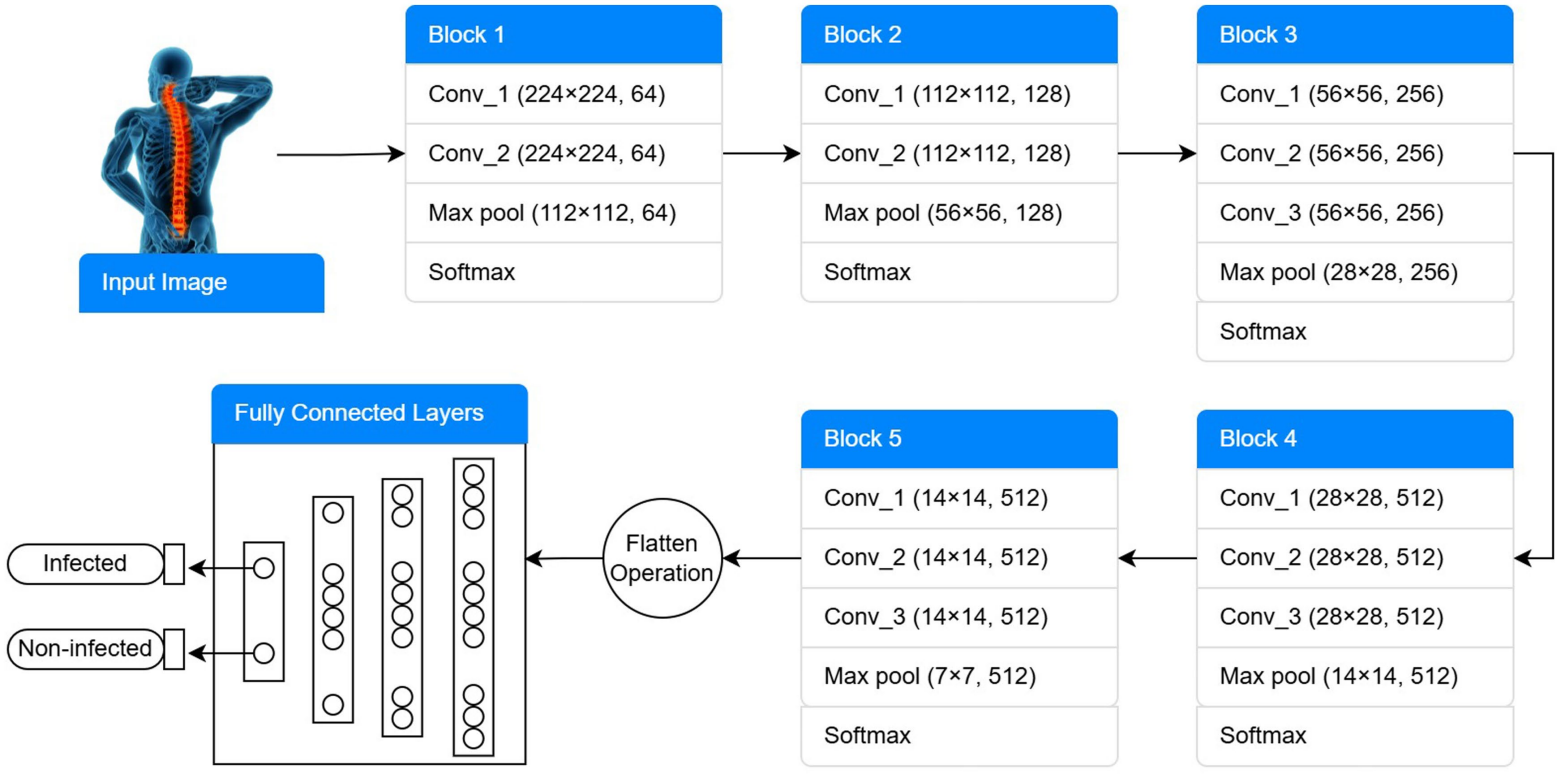
Architectural schematic of the VGG-16 transfer learning model used for spine DXA classification.

DXA was considered the standard examination for BMD valuation (see Figure 1). Elderly patients with multiple diseases are increasing day by day overall in the world, and mostly with bone fracture diseases; their examination DXA scans are highly utilized. Sometimes, vertebral fractures are detected incidentally, without serious symptoms, during the evaluation of other diseases. BMD can be estimated from a DXA scan, which helps physicians adopt appropriate strategies to prevent fractures and benefit patients. The World Health Organization (WHO) criteria for diagnosing osteoporosis are based on the T-score from the DXA scan dataset (Sultana et al., 2024; Uemura et al., 2024).

The novelty of this study lies in diagnosing osteoporosis using a deep learning method to analyze BMD values from DXA scans. To detect spine osteoporosis in DXA scan images, we developed a specialized 21-layer convolutional neural network (CNN). The primary objective of this study is to accurately diagnose osteoporosis, enabling our experts to identify fracture patterns associated with the condition. To the best of our knowledge, no previous research has used a CNN to characterize spine osteoporosis based on BMD values and DXA images. Additionally, it reduces the burden on radiologists, who must use a variety of techniques to identify fractures in the spine. Additionally, the proposed model is compared with four key medical classifiers: Visual Geometry Group 16 (VGG-16), VGG-19, and InceptionV3.

The main contribution of this study is stated as follows:

- Developing a 21-layer CNN model that automatically detects osteoporosis using information from spine DXA images.
- With a 97.16% F1-score, 95.41% recall, 99.04% precision, and 97.14% accuracy, the model outperformed baseline pre-trained models.
- To detect osteoporosis in the spine, a novel deep learning algorithm was created. This reduced the time radiologists needed to manually examine the image.

The rest of the article is structured as follows: Section 2 provides an overview of the literature review. Section 3 addresses data structure, description, and preparation. Section 4 contains experimental results and discussion. Section 5 details the investigation’s outcomes.

## 2 Related works

Given that numerous diseases lead to reduced bone density, this study showed that visual screening for osteoporosis using computed radiography (CR) images is a useful technique, and an automated method using CR image datasets to detect osteoporosis. To distinguish between damaged and undamaged images in the dataset, a deep convolutional neural network (DCNN) classifier is first used. The convolutional neural network is trained and evaluated using pseudo-images. Approximately 64.7% of the results were produced using this approach (Williams et al., 2024; Schröder et al., 2024). This work presents a novel U-Net architecture with side layers and a consideration unit to address the challenging task of detecting osteoporosis using DXA and radiographic images. This design enables proper segmentation of bone components. A computerized method based on phalangeal computed radiography (CR) images is used to identify osteoporosis. The suggested method uses a deep convolutional neural network (DCNN)-based classifier to distinguish between normal and aberrant CR pictures (Goller et al., 2023). Like CNN, pseudo-color visuals are used for evaluation and instruction. Finite Element Analysis (FEA)-based biomechanical modeling tools have shown promise in recent years for better medical judgment. To create a complex mathematical model involving partial differential equations that can only be solved by hand, these methods require geometrical data from medical imaging, patient background history, and brief patient information (Bourigault et al., 2024). This method is computationally intensive and best suited to algebraic equations. Building high-fidelity models that accurately depict the entire three-dimensional (3D) space can take hours or even minutes, as FEA relies heavily on CT imaging data, often referred to as biomechanical CT (BCT). The CNN method was used to reduce the number of patients and to computerize the results of bone CT scans (Mallio et al., 2024). The strategy incorporates Imprint Division, Bone Con ioV, an organization whose market division objective is to optimize the location and return on the initial capital investment segment (locale of premium division), utilizing the bone-conditions-classification-network (BCC-Net) group stepping-stone one through features. The pre-arranged MS network provides a stepping stone for input CT images, which prompts division, and the BCC network identifies probable organizations, Osteopenia, and bone fractures by partitioning the input CT (Bott et al., 2023). The results of these organizations help radiography experts with the basis of quality investigations of bones. A performing approach is used in a Deep Neural Network to process ImageNet images for DPR images and to improve accuracy in analyzing osteoporosis. The Alex-Net architecture is utilized for tweaking and information (patient) classification, with numerous intermediate fixes, including the removal of DPR pictures, and the trained components (dental information) are used for osteoporosis localization (Najafi et al., 2023). Along these lines, the Octuplet Siamese Network (OSN) is the highest performing component and the eighth most advanced in terms of return on capital invested in DPR image classification. The cross-approval is left to accomplish higher exactness (He et al., 2024). The X-ray and Fracture Risk Assessment (FRAX) tool provided the most effective results for detecting bone fractures. The improvement in bone quality through Artificial Neural Network (ANN) planning. Expansion of Teriparatide (TPD) synthesis for not just renovating Bone Mineral Thickness, but additionally Bone–Strain–File, Trabecular Bone Score, which reinforces the bone and limits the danger of fracture (Nicolaes et al., 2024). The Bone Strain Index (BSI) is, by all accounts, a measure of the TPD’s effectiveness. ANN is a legitimate tool for clinical examination. ANN is superior to calculated relapse (logistic regression [LR]) in terms of precision, as it uses a larger dataset, discrete boundaries, and an optimal procedure for analyzing osteoporosis. The aftereffect of ANN concerning the Area under the curve is more than LR (Liu et al., 2024).

Fuzzy logic calculations can be used with LR, which is the best scientific technique and is established as a standard practice. To coordinate with the data, the executive’s framework for better investigation. The ANN product bundle is a proficient methodology for precise segmentation and BMD estimation (Öziç et al., 2023). The information includes: weight, sex, age, body mass index (BMI), and tallness adjacent to with segmental reference, and BMD (total values) are taken care of by ANN (complex info layer), and a quantifiable estimate of (bone mineral density of legs, arms, pelvis, spine, and total) is produced in the yield layer. The Artificial Neural Network model presents a possible methodology for assessing BMD (absolute qualities) and, segmentally, utilizing measurable qualities, demonstrating the finest model for identifying osteoporosis. To distinguish Osteoporosis, a motivation reaction test was performed on the tibial bone using LabVIEW. The record of the simple sign was concentrated in frequency space (Geetha et al., 2024). The vibration produced by regular recurrence significantly decreased osteoporosis, indicating a decline in bone strength and bone mineral mass. In a new report, osteoporosis was discovered to be costly and necessary for the virtuoso apparatus (Du et al., 2025). The technique used in the review was easy to use and less expensive. The pattern of simulated intelligence—machine learning (ML) exertion connected to the spine incorporates vertebral confinement and images of radiographs (discs) region of interest (ROI), computer-aided design, clinical practice forecasting and troubleshooting, data management, biomechanics, recovering the content of images, and motion study. The use of artificial intelligence in Clinical science gathers and confirms information, providing the security of local area genuine space apparatuses (Shen et al., 2023). Classifiers in machine learning, as implemented in Waikato Environment for Knowledge Analysis (WEKA—a benchmark for ML), are tested using 10-fold cross-validation, prepared datasets, and feature selection and extraction (Zhang et al., 2025). The correlation of the outcome is performed in terms of execution time, characterized occurrences, mean absolute errors, and kappa insights assessment, consideration, and rejection of component determination (Kim et al., 2023). The general review proposes better outcome prohibition, including determination, where these strategies, Instance-Based for K-Nearest Neighbor (IBK), Logistic Model Tree (LMT), J48, JRip, Sequential Minimal Optimization (SMO), and bagging, give an apparent effect of incorporation highlight choice. Surface portrayal of healthy bone is essential for osteoporosis (Zhang et al., 2025). The Gray-Level Co-Occurrence Matrix (GLCM), Local Binary Pattern (LBP), laws, etc., are standard techniques used for surface element extraction, bridging the gap between deep component extraction from CNN and ordinary elements (Shams Alden and Ata, 2025). The results of this review indicate that profound elements have a significant impact on the classifier’s performance, consistently outperforming its performance on regular elements (Chen et al., 2023).

To distinguish between osteoporotic and osteopenic states, the current work demonstrates how to incorporate recently acquired data on extremely low-power radiofrequency delivered via the wrist using a neural organization classifier. They divide the acquired data into two binary categories using our methods (Asamoto et al., 2024). With a Double X-ray Absorptiometry (DXA) T-score of less than −1, 27 osteoporotic/osteopenic patients with low bone mineral density (BMD) were included in Gathering 1. They were predicted to live for a year. A total of 40 healthy participants, the majority of them being young, who did not have significant clinical risk factors, such as a (family) history of bone fractures, made up Gathering 2 (Requist et al., 2024). They discovered a perplexing radio frequency (RF) spectrum spanning from 2 GHz to 30 kHz. Measuring the two wrists separately from the wrist circuit and then integrating the findings significantly improves accuracy compared with averaging the data from the two wrists (Alavi et al., 2024). It takes less than a minute to estimate and get data. The neural organization classifier achieves 83% affectability and 94% specificity for the RF range. Significance: These findings were obtained without the use of any novel clinical risk variables (Namatevs et al., 2023). They demonstrate that radio transmission data is the only reliable method for estimating bone thickness.

Whether or not medical information is provided, this study demonstrates how the authors utilize hip radiographs to diagnose osteoporosis and perform an indicative analysis in image mode (Najafi et al., 2023). This research aimed to collect 1,131 images of patients who underwent hip radiographs and skeletal BMD testing in the same year between 2014 and 2019. To identify osteoporosis in hip X-rays, five CNN models were employed. Clinic variables are added to an equal number of models in each CNN. Each organization was evaluated based on its AUC, F1-score, explicitness, review, exactness, accuracy, and negative predictive value (NPV). Using only hip radiographs, the researchers compared five CNNs and found that GoogleNet and EfficientNet B3 were the most accurate and effective. EfficientNet B3 achieved the highest accuracy, F1-score, NPV, and AUC when patient characteristics were taken into account (Ong et al., 2023). A convolutional neural network model is used to identify osteoporosis in hip radiographs, according to a high-quality evaluation study. The findings align with the clinical indicators noted in the patient record. To cluster images of osteopenia and osteoporosis, this study employed a DCNN model based on Lumbar Spine X-rays (Yen et al., 2024). The receiver operating characteristic (ROC) curve analysis technique was used to evaluate the model’s performance. In test dataset 1, the model with an AUC of 95% and an accuracy of 73.7% outperformed the model with an AUC of 0.787 and an accuracy of 81.8% (Hung et al., 2024). In test dataset 2, the osteoporosis detection model achieved an AUC of 0.726 and an accuracy of 68.4%, whereas the osteoporosis identification model yielded an AUC of 0.810 and an accuracy of 85.3%. Table 1 presents some state-of-the-art methods and their corresponding results.

**Table 1:** Comparison of prior studies.

Ref	Model	Dataset	Classifiers	Accuracy (%)	AUC (%)	Sensitivity	Specificity
Sollmann et al. (2022)	DCNN	X-ray images of dental panoramic	SC-DCNN	98% & 98.5%	97.63 99.91 99.87%	0.89%	0.957%
Liu (2024)	CNN	CT scans of the lumbar vertebra	MS-Net BCC Net	76.5%	91.67%	-	-
Senanayake et al. (2023)	Multi-task	X-rays dental panoramic	OS-Net	0	-	-	-
Duraivelu et al. (2023)	ANN	X-ray images	LSTM CNN	78.8% 78.3%	-	-	-
Jang et al. (2022)	ANN	X-ray images Vertebral	ANN	95.8%	0.950% 0.870%	94.5%	96.9%
Oh et al. (2024)	ANN	DXA Arm, leg, spine, pelvis, total	ANN	99%	-	-	-
Peng et al. (2024)	Multiple regression	CT scans	Logistic regression	92.5%	90%	0.969%	0.939%
Feng et al. (2024)	GLCM	X-ray images Calcaneus	SVM NNs	95%	-	50%	50%
Pan et al. (2024)	CNN	MRI	117 epochs	92.5%	1.000	75%	97%

## 3 Materials and methods

### 3.1 Cohort characteristics

This study uses the publicly available spine DXA dataset (Kale et al., 2025), which contains DXA scan information from 700 Indians aged 25–85, with 350 men and 350 women. The collection includes measurements such as BMD, T-score, Z-score, BMI, obesity group, body fat percentage (BFP), and soft tissue density (STD). Our lab-studied 174 DXA scans, including 60 (age = 62.3 ± 7.8, BMI = 21.4 ± 2.8) from adults who had never broken a bone and 114 (age = 61 ± 8.4, BMI = 22.7 ± 3.1) from those with a history of minor fractures. Table 2 offers a thorough explanation of the DXA femoral neck dataset. The sample sizes, demographics, gender distributions, BMD, T-scores, Z-scores, and fracture histories are provided for both the osteoporosis and normal groups.

**Table 2:** Summary of the dataset used.

Class	BMD (mean ± SD, g/cm^2^)	Male/Female	Z-score (Mean ± SD)	Age (Mean ± SD)	BMI (Mean ± SD)	Number of samples	T-score (mean ± SD)	Fracture history
Normal	0.95 ± 0.12	30/30	0.1 ± 0.1	62.3 ± 7.8	21.4 ± 2.8	60	−0.8 ± 0.2	No
Osteoporosis	0.62 ± 0.1	55/59	−0.5 ± 0.2	61 ± 8.4	22.7 ± 3.1	114	−2.9 ± 0.3	Yes

### 3.2 Data pre-processing and augmentation

The spine DXA scans employed in the research measured 690 × 1,340 pixels in height and 1,350 × 2,800 pixels in width. Images were reduced to 300 × 300 pixels for pre-trained models (VGG-16, VGG-19, ResNet-50, and InceptionV3) and 150 × 150 pixels for the proposed 21-layer CNN to standardize the dataset for CNN input. Pixel intensities were adjusted during training to promote model convergence. We focused on the area of interest (ROI) that matches the vertebral bodies most affected by osteoporosis rather than the full image. Skilled radiologists methodically reduced the ROI and identified spinal sections based on BMD trends and anatomical parameters. This strategy assured that the model learns features from clinically significant areas while reducing background noise. All ROI annotations were standardized to provide uniformity and consistency throughout the collection. Segmentation networks for autonomous ROI extraction could be applied in future research to increase the efficiency of healthcare procedures.

To increase the adequate dataset size and reduce overfitting, multiple data augmentation techniques were applied exclusively to the training set:

- Width and height shifts: ±0.2% of the image dimensions to simulate horizontal and vertical translations.
- Rotation: random rotations up to ±40°.
- Shear transformation: 0.2% anticlockwise to simulate perspective variations.

By combining ROI-focused pre-processing with these augmentation strategies, the model learned robust features from relevant vertebral regions while improving generalization performance. The complete list of augmentation parameters is summarized in Table 3.

**Table 3:** Parametric values for data augmentation.

Parameters	Value
Width and height shift	0.2%
Horizontal flip	True
Vertical flip	False
Shear	0.2%
Zoom	0.2%
Rotation	±40°

Although the original DXA images range from 690 × 1,350 to 1,340 × 2,800 pixels, we resized them to 150 × 150 pixels for our proposed CNN model to reduce computational load and enable efficient training. Prior studies have shown that CNNs can capture sufficient global and structural features for BMD-based classification even at lower resolutions, as fine-grained trabecular patterns often manifest as textural and intensity differences across regions. Additionally, data augmentation and the multi-layer convolutional architecture of our model help preserve critical spatial features, mitigating potential information loss from down-sampling.

### 3.3 Transfer learning image classifiers

Prominent state-of-the-art transfer learning classifiers, such as ResNet-50, InceptionV3, VGG-16, and VGG-19, are included in this category. These classifiers were used in clinical settings to distinguish radiographs with osteoporotic damage from those without fractures. The ImageNet Large-Scale Visual Recognition Challenge (ILSVR) database, which contains hundreds of different object categories for model training and classification performance testing, was used to develop these classifiers. VGG-16 is widely used across research applications due to its open-source nature. There are six stages in the VGG-16 pre-trained model architecture. Two strides, “two convolutional layers,” and “one max-pooling layer” are included in the first two stages. The next three phases comprise three convolutional layers, one max-pooling layer, and two strides. VGG-16’s third and final phase replaces the fully connected layer (FCL) with a sigmoid activation, giving the model three fully connected layers and the ability to identify osteoporosis in radiographic images. Compared to VGG-16, VGG-19 features a more complex pre-trained model architecture and a higher training cost. A residual network with 50 layers and four stages is referred to as ResNet-50. Three deep residual networks with kernel sizes of 1, 64, 64, and 256 were used for this task. Many people classify illnesses using CNN-based inception models. You may train on millions of datasets from the ImageNet collection using InceptionV3. These four pre-trained classifiers are widely used by academics and are acknowledged as the most advanced for image classification in the medical field.

Visual Geometry Group (VGG-16 and VGG-19): For this research, the ImageNet (ILSVRC) database is trained using a pre-trained deep convolutional neural network architecture. The database consists of several object classes for evaluating and training image classification models. An open-source VGG-16 framework has been developed to diagnose osteopenia on spine radiographs and is also used in many other research studies. Using the sigmoid function, the architecture of VGG-16’s final three layers was replaced to permit the model to diagnose osteoporosis. Furthermore, there’s another framework, VGG-19, which is deeper than the VGG-16 pre-trained model. VGG-19, compared to VGG-16, is more computationally intensive during network training and has more trainable parameters. The VGG-16 schematic block diagram is given in Figure 1. Figure 1 shows the architecture of a pre-trained VGG-16 model used to detect spine DXA pictures. The final model comprises 13 convolutional layers with Rectified Linear Unit (ReLU) activations, five max-pooling layers, and three fully connected layers (FCL). The final layer employs sigmoid activation to differentiate between the normal and osteoporosis classes.

**Figure 2:**
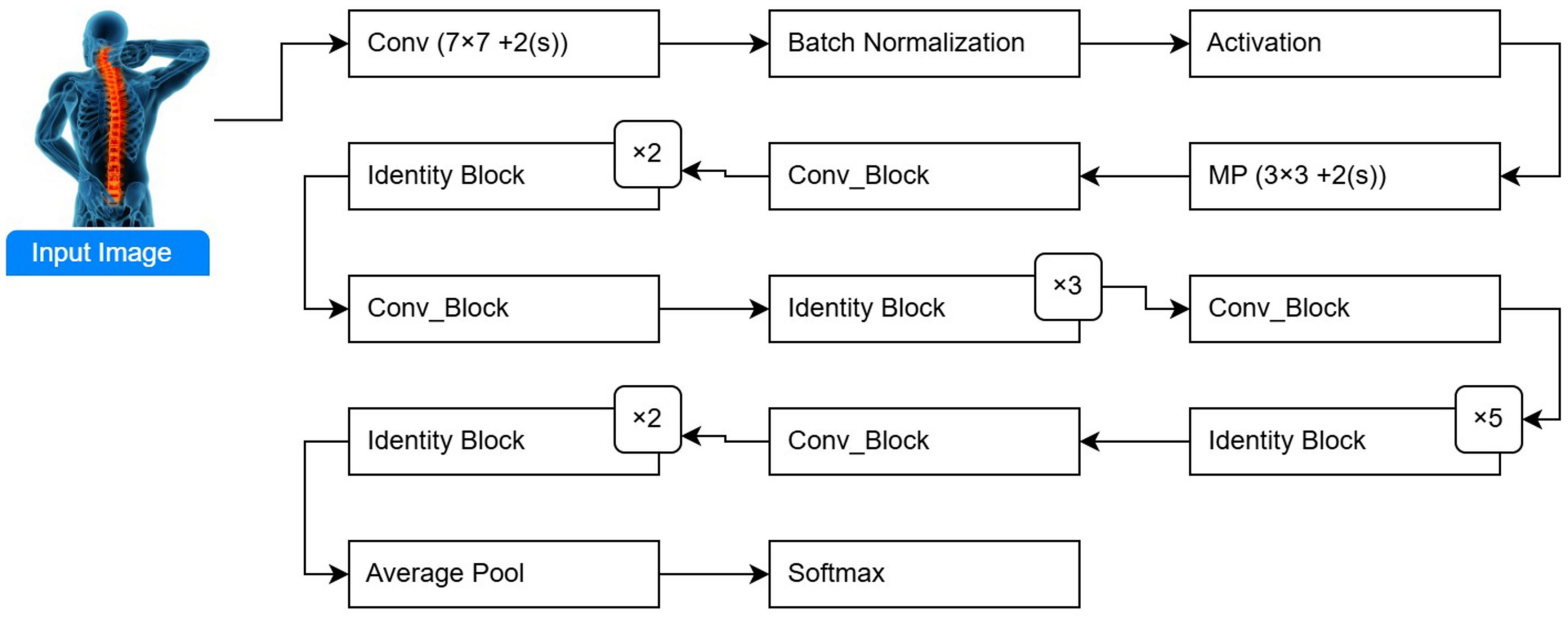
Transfer learning architecture of the ResNet-50 model applied to spine DXA images.

ResNet-50: To achieve strong convergence behavior, some network layers of the designed ResNet model are passed through using a skip connection. ResNet-50 is called the enhanced version of ResNet. Although ResNet and VGG-Net share a similar architecture, ResNet is approximately eight times deeper. Figure 2 shows the transfer learning architecture of the ResNet-50 model. The model includes 50 layers and residual connections to increase gradient flow and deep network training for osteoporosis diagnosis using spine DXA images.

**Figure 3:**
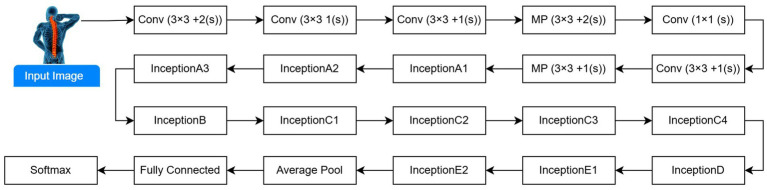
InceptionV3 theoretical framework adapted for DXA image classification.

InceptionV3: For medical image classification, a CNN-based inception model is mainly used. Inception is an alteration of InceptionV3, utilizing millions of images derived from the ImageNet database for training. The updated InceptionV3 model architecture for spine DXA categorization is shown in Figure 3. The model uses multiple convolutional kernels of varying sizes in each block to effectively capture spatial input. After pre-training on ImageNet, the DXA dataset was refined for osteoporosis diagnosis. Pre-trained on ImageNet and fine-tuned on the DXA dataset for osteoporosis detection. The reason for selecting the ResNet-50, VGG-16, VGG-19, and InceptionV3 models is that they are widely used for medical classification and recognition. Depending on the pre-training, large-scale ImageNet database models have fixed weights, except for the FCL, which was initialized randomly. Moreover, with a fine-tuning version of a pre-trained network, the model’s weights were initialized using the ImageNet database of previously trained models, at every step of training, except for the final blocks, which were unfrozen, allowing their weights to be updated.

### 3.4 Proposed CNN architecture

To extract the most important and dominant characteristics, a specialized CNN model with 21 layers was constructed. The CNN model was trained using RGB images (150 × 150 × 3). There are three channels ( $n_{c}$ ) in the input image. The suggested 21-layer CNN architecture strikes a balance between dataset size and model complexity. The network’s layer count was adjusted to aggregate edges, textures, and structural patterns associated with osteoporosis by gradually aggregating low-, mid-, and high-level features from spine DXA images. To enable the network to learn more abstract representations while preserving spatial information in the early stages, filter sizes and a scaling method (beginning with 32 filters and expanding to 512) were used. Dropout layers (rate = 0.2) were used to avoid overfitting, and max-pooling layers were added to reduce the size and computational expense of the feature map. The design’s major goal was to maximize feature extraction from limited ROI areas while preserving efficient training and generalization.

The dimensions of the filter are ( $f_{h}\;$ × $\;f_{w}$ ). The filter, also referred to as a kernel, has three widths and values. Generally, the height ( $f_{h}$ ) and the width ( $f_{w}$ ) of the filters remain the same. “Feature identifier”’ is another name for this filter. Low-level features, such as edges and curves, can be obtained by layering using these filters. To improve deep feature extraction from images, more convolutional layers are added, enabling the model to capture the full image characteristics. Table 4 presents a CNN model that explains the step-by-step addition of more convolutional layers. In the sub-area of the image convolution operation is performed using the filter. In a convolutional operation, the image pixel value and the filter are multiplied and aggregated elementwise. The parameter weight corresponds to the filters’ values. Training models learn these weights. At the beginning of the image, the filter starts the convolution. By covering the entire image, it continuously shifts across the image by a fixed unit amount to execute the convolution, producing a single value and a single operation output.


$$C=I\times F=\sum \sum I(i+m,j+n)F(f_{h}*f_{w})\;\;\;\;$$ (1)

**Table 4:** The detailed description of the proposed CNN architecture.

Layer	Pool size	Filter size	Stride	Number of filters	Dropout	Activation
Conv2D	–	3 × 3	1	32	–	ReLU
Conv2D	–	3 × 3	1	64	–	ReLU
Conv2D	–	3 × 3	1	128	–	ReLU
MaxPooling 2D	2 × 2	–	2	–	–	–
Dropout	–	–	–	–	0.20	–
Conv2D	–	3 × 3	1	64	–	ReLU
MaxPooling 2D	2 × 2	–	2	–	–	–
Dropout	–	–	–	–	0.20	–
Conv2D	–	3 × 3	1	128	–	ReLU
MaxPooling 2D	2 × 2	–	2	–	–	–
Dropout	–	–	–	–	0.20	–
Conv2D	–	3 × 3	1	512	–	ReLU
MaxPooling 2D	2 × 2	–	2	–	–	–
Dropout	–	–	–	–	0.20	–
Conv2D	-	3 × 3	1	512	-	ReLU
MaxPooling 2D	2 × 2	–	2	–	–	–
Dropout	–	–	–	–	0.20	–
Flatten	–	–	–	–	–	–
FCL	–	–	–	64	–	ReLU
Dropout	–	–	–	–	0.20	–
FCL	–	–	–	2	–	Sigmoid

A convolution operation is described by Equation 1, where the * operator denotes the process and F denotes the input and filter sizes ( $f_{h}*f_{w}$ ). The amount of shift applied to the filter is controlled by the stride parameter. The model’s convolutional layers all have a stride of 1. The input volume’s width and height decrease as the number of strides rises. Issues such as the receptive field exceeding the input volume and dimension contraction may arise when the stride values for the receptive field’s minimum overlap is high. To mitigate this problem, “zero padding” is used. The input is padded with zeros at the borders to maintain consistency in the volume dimension with the output. The size of the zero padding can be determined by Equation 2 if having a stride size of 1:


$$\text{Zero padding}=\frac{f-1}{2}\;\;$$ (2)

This feature ensures that the filter’s width and height are identical. The suggested 21-layer model uses valid padding instead of zero padding, which reduces the output dimension after convolution so that it differs from the input dimension. Several properties are taken out. There are several filters in the convolutional layer. Thirty-two filters were used in the first layer; subsequent layers increased the number of filters from 32 to 128 to 512, and so on. Another name for the output value is “activation map” or “feature map.” Equations (3–5) determine the form of each output layer.


$$O_{h}=\frac{I_{h}-f_{h}+2P}{S}+1\;\;$$ (3)

$$O_{w}=\frac{I_{w}-f_{w}+2P}{S}+1\;\;\;\;$$ (4)

$$o_{n}=N_{f}\;\;$$ (5)

Equations (3–5) represent the input height by $I_{h}$ , input width by $I_{w}$ , $f_{h}$ is filter height, $f_{w}$ shows the filter width, the stride size is defined by the *S*, *P* is the padding, and $N_{f}$ represents the filter number. The values of the first layer of our proposed model ( $I_{h}$ = 150, $I_{w}$ = 150, $f_{h}$ = 3, $f_{w}$ =3, *S* = 1, *p* = 0, and $N_{f}$ = 32) are calculated by Equations (6–8).


$$O_{h}=\frac{150-3+0}{1}+1=148\;\;\;\;$$ (6)

$$O_{w}=\frac{150-3+0}{1}+1=148\;\;$$ (7)

$$O_{n}=32\;$$ (8)

The convolutional layer performs linear computations, such as element-wise summation and multiplication. To introduce non-linearity into the linear operation of the convolution layer, the ReLU activation is applied. Equation 9 shows the ReLU operation function:


ReLU(X)=max(0,X) (9)

The result of a convolution is denoted by X. Converting all negative outputs to zero is the aim of ReLU to improve computation speed and increase the accuracy of the suggested model in detecting non-linearity. The issue of fading gradients is lessened at lower levels when the layers are trained gradually.

**Figure 4:**
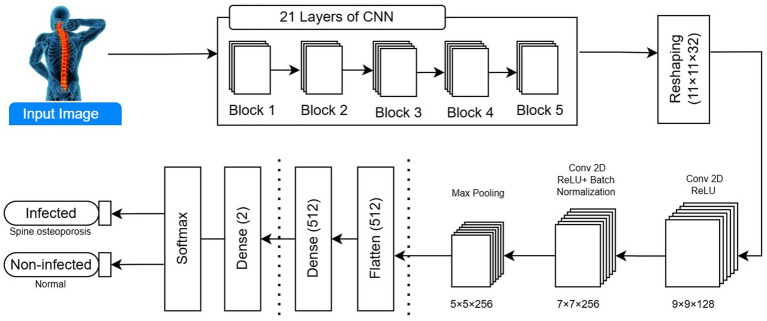
Proposed 21-layer CNN architecture for automated osteoporosis detection from spine DXA scans. The model includes sequential convolutional layers with increasing numbers of filters, max pooling for spatial down-sampling, dropout for regularization, and fully connected layers for final classification.

A max-pooling layer followed the two convolutional layers. This layer reduces the width and height of the supplied image. As shown in Figure 4, CNN’s suggested model has a stride of two and a 3 × 3 filter size. The general architecture of the proposed 21-layer CNN for detecting osteoporosis from DXA spine data is shown in Figure 4. The model uses convolutional layers with increasing filter sizes (32 to 512), max-pooling layers for down-sampling, dropout layers (rate = 0.2) to avoid overfitting, and fully connected layers for classification. In conclusion, 150 × 150 × 3 input images are separated into osteoporosis and normal classes. To extract the maximum value from the relevant field, the filter convolves around the input volume. Analyzing a feature’s relative position rather than its actual location is the most effective approach for this layer. Removing overfitting and reducing the weights, computation costs. The dropout layer is then used. A few activation functions in this layer were deliberately reset to zero. Even if partial activation is lost, this layer ensures that the model accurately predicts the image’s categorization. Consequently, the dataset should not be used to train the model. Overfitting may be prevented by removing the layers. The dropout layer threshold is 0.20. The flattened layer is then passed to a fully connected layer (FCL) after being reduced from a 2D feature map to a 1D feature vector. Until the image’s deep qualities are recovered. FCL uses a one-dimensional feature vector to classify osteoporosis. There are 64 neutrons in the anticipated CNN’s FCL. The model can distinguish between events with and without osteoporosis class labels when softmax is enabled in the output layer. The first FCL alerts the second FCL to the output activation.

## 4 Experimental results

For training, the model and randomly divided into five folds, and spin radiograph images were selected. To estimate the model, a 5-fold cross-validation (CV) was performed to prevent overfitting.

Several precautions were taken to prevent the risk of overfitting, considering the small dataset size (174 DXA images). After 10 consecutive epochs in which the validation loss did not decrease, training was terminated early. To improve generalization and decrease neuron co-adaptation, L2 regularization and dropout layers (rate = 0.2) were used. All pre-processing and augmentation operations were limited to the training set to prevent data leakage into the validation or test sets. These strategies significantly reduced overfitting while maintaining the robustness of the suggested CNN model during five-fold cross-validation.

The dataset was split into training and test sets, with 70% allocated to training and 30% to testing. Compared with other training folds, the validation set was a distinct fold and was used during training to assess the model’s performance. After completing one step, the individualistic fold was used for model training as a validation set, and the previous validation set was also used again as a segment of the training set to calculate the model training.

To improve model training and adjust hyperparameters, 5-fold cross-validation (CV) was performed on the training dataset. Each of the remaining folds was used as a validation subset once during training. The test set was used solely for the final performance assessment and was excluded from cross-validation. By stabilizing training and limiting overfitting on the training set, 5-fold CV ensures that the metrics accurately reflect the model’s capacity to generalize to new data.

The collection, which contains 60 normal scans and 114 osteoporotic pictures, displays a substantial class imbalance. We added class weighting to the loss function, giving the underrepresented normal class greater weight to prevent bias toward the dominant class. Furthermore, to improve effective sample sizes and sustain model convergence, data augmentation was appropriately applied to each class. Although class weighting was an obvious and cost-effective choice, other strategies, such as attention loss or oversampling of the minority class, were also investigated. These techniques reduced bias in predictions while ensuring that the model acquired discriminative characteristics from both groups.

The model was trained with a batch size of 16, an initial learning rate of 0.001, and the Adam optimizer, and with Keras’ default weight initialization for convolutional and fully connected layers. Grid search was used to run experiments with different learning rates to maximize the trade-off between convergence speed and generalization. Classification was achieved using cross-entropy loss and the softmax activation function of the output layer. To guarantee consistency, all training folds and pre-trained models were assigned to the same hyperparameter values.

An internal train-test split (70% training; 30% testing) and 5-fold cross-validation were used to evaluate the proposed model; however, no independent external validation cohort was employed. We were unable to validate on another dataset due to the limited availability of DXA spine images. As a result, the present findings concentrate exclusively on the model’s internal generalization efficiency within the same dataset. Future work will leverage unique, multi-center datasets from a larger, more diverse patient population to test the clinical applicability and generalizability of the proposed CNN model across a range of imaging settings.

**Figure 5:**
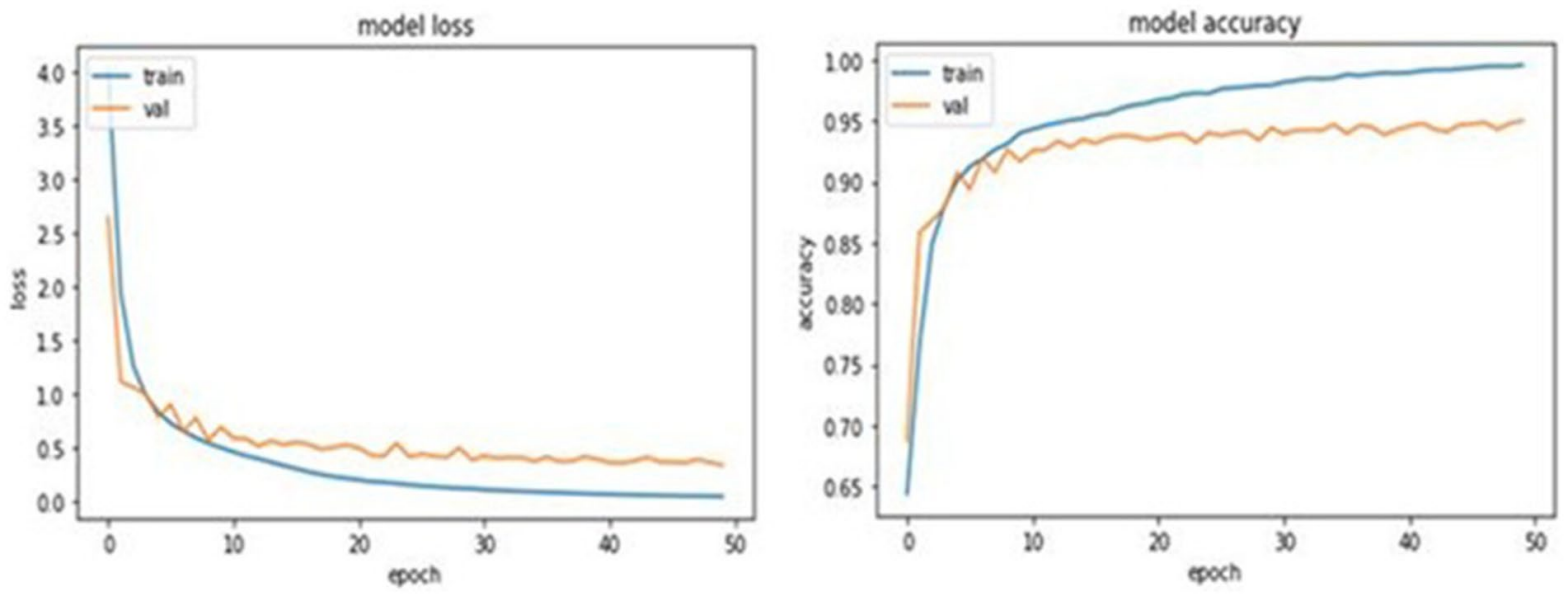
Training and validation performance of the proposed 21-layer CNN model over 50 epochs.

Image features and attributes are used in the suggested 21-layer CNN approach to detect osteoporosis. Up to 50 epochs are used to train the 21-layer CNN model. The F1 score, confusion matrix, recall, precision, and ROC curve were used to evaluate the classification performance of the suggested CNN model. Figure 5 shows the training and validation accuracies of the proposed 21-layer CNN model over 50 epochs. The training accuracy of 0.99 and the validation accuracy of 0.96 indicate strong learning and low overfitting. Loss curves indicate ongoing convergence during training. The 21-layer CNN model was evaluated throughout 50 epochs. Training and validation had the highest accuracy scores (0.99 and 0.96, respectively). Training and validation accuracy and loss were assessed over 50 epochs of the proposed 21-layer CNN model.

The proposed model achieved training and validation accuracies of 0.99 and 0.96, respectively, indicating excellent learning capability. The small gap between training accuracy and validation accuracy is expected, given the model’s depth and the limited dataset size. Several regularization techniques were used to reduce overfitting during training. Dropout layers (rate = 0.2) were used to prevent neuron co-adaptation, and excessive weights were penalized with L2 weight regularization. Moreover, when the validation loss stopped improving, training was stopped early after 10 epochs. Grid search was used to examine hyperparameters, including learning rate, batch size, and optimizer settings, to find the best trade-off between model complexity and generalization performance. The combination of these tactics may have contributed to minimal accuracy shifts throughout training and validation by keeping the model well-regularized.

The experiment’s results demonstrate how well our system learns to distinguish osteoporosis from other conditions. In training, the loss was 0.081, and in validation, it was 0.09. The diagnostic model for osteoporosis was evaluated using multiple performance criteria. The confusion matrices for the CNN model, which uses four pre-trained CNN classifiers and 21 layers, are shown in Figure 2. There are 105 normal and 105 infected DXA images in the testing set. Confusion matrices are composed of expected cases in the columns and actual occurrences in the rows. Of the 105 normal instances, the suggested method accurately identified 104, but it misidentified one as osteoporosis.

**Figure 6:**
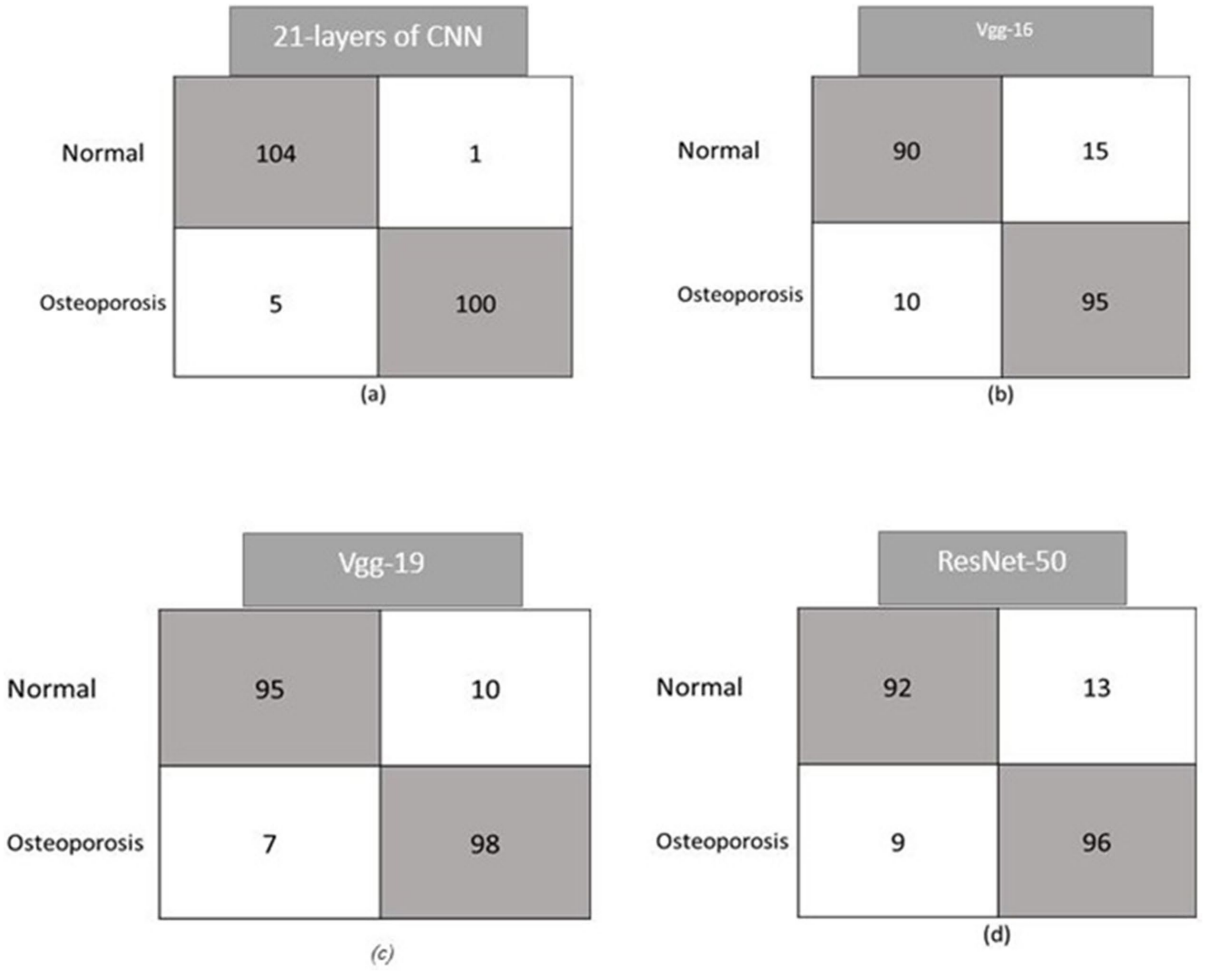
Confusion matrices for the proposed CNN and four pre-trained classifiers (VGG-16, VGG-19, ResNet-50, InceptionV3).

In a similar vein, the algorithm incorrectly identified five instances as normal while predicting 100 occurrences as osteoporosis when given an osteoporosis class. The model forecasts 90 instances of VGG-16 occurring normally and 15 cases of osteoporosis with incorrect labels. According to the model, 95 of the 105 people would have osteoporosis, while 10 will be OK. Ten people are incorrectly classified as having osteoporosis by Resnet-50, which correctly forecasts 95 out of 105 cases as normal. In a similar vein, the model incorrectly identified 98 cases as osteoporosis while misdiagnosing 7 patients as normal. Nine of the 105 osteoporosis cases were misclassified as normal by the VGG-19 model. The software identified 92 occurrences in healthy individuals and mistook 13 cases for osteoporosis. The confusion matrices for the osteoporosis and normal classes, including precision, recall, accuracy, and F1-score, are shown in Figure 6 and presented in Table 5. For the test dataset, Figure 6 shows the proposed CNN alongside the confusion matrices of the four pre-trained classifiers (VGG-16, VGG-19, ResNet-50, and InceptionV3). The number of spine DXA images that were accurately and incorrectly classified as normal or osteoporosis is shown in each matrix. The recommended CNN has the highest accuracy rate in both courses.

**Table 5:** Performance evaluation of the proposed model with state-of-the-art classifiers.

Model	Accuracy (%)	Precision (%)	Recall (%)	F1-Score (%)
VGG-16	88.09	85.71	90	87.8
VGG-19	89.52	87.61	91.08	89.35
ResNet-50	91.9	90.47	93.13	91.77
InceptionV3	84.76	83.8	85.43	84.62
Proposed model	97.14	99.04	95.41	97.16

To determine if the performance differences between the suggested 21-layer CNN model and the baseline pre-trained classifiers (VGG-16, VGG-19, ResNet-50, and InceptionV3) were significant enough to warrant comparison, statistical significance testing was conducted. For each of the five cross-validation folds, the classifiers’ mean accuracy and standard deviation were calculated. A paired *t*-test was then used to evaluate the accuracy of the proposed model against each baseline model. The suggested CNN model significantly improved performance (*p*-values < 0.05). F1-scores and 95% confidence intervals were used to assess the accuracy of the data that was supplied. These statistical tests confirm that the claimed improvements in performance are real and not due to random variation.

The 21-layer CNN model achieved 99.04% accuracy, 95.41% recall, and 99.04% precision with an F1 score of 97.16%. With 85.71% accuracy, 90% recall, and 85.71% precision, the VGG-16 received an F1 score of 87.80%. With a precision of 90.47%, a recall of 93.13%, and an accuracy of 91.90%, ResNet-50 achieved an F1 score of 91.77%. With an F1 score of 89.35%, the VGG-19 model achieved 89.52% accuracy, 91.08% recall, and 87.61% precision. InceptionV3 achieves 84.76% accuracy, 85.45% recall, and 83.38% precision, with an F1 score of 84.62%. The ROC curve is created by plotting the false positive rate (FPR) on the y-axis and the true positive rate (TPR) on the x-axis.

**Figure 7:**
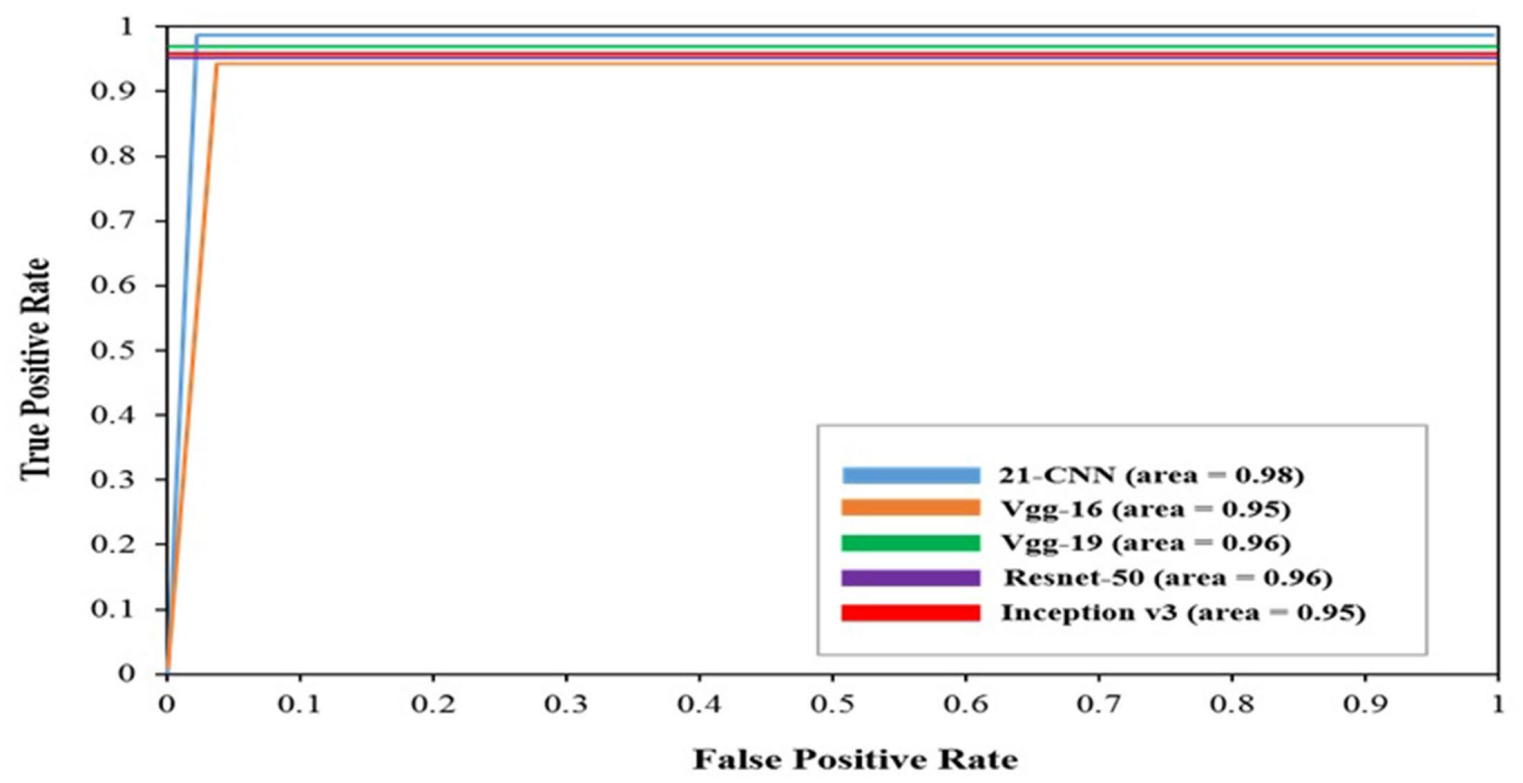
Receiver operating characteristic (ROC) curves for the proposed CNN and baseline classifiers.

In medical diagnosis, the model is considered more efficient if its ROC curve has a higher area under the curve. Figure 7 shows the ROC curves of our classifier. For the 21-layer CNN, the AUC was 0.9823. For VGG-16, the AUC was 0.9580. For VGG-19, the AUC was 0.9599. For Resnet-50, the AUC was 0.9626. For InceptionV3, the AUC was 0.9583. Thus, in detecting osteoporosis cases from spine DXA images, the model could make an efficient contribution. Receiver operating characteristic (ROC) curves for the four pre-trained classifiers and the suggested 21-layer CNN are shown in Figure 7. AUC values demonstrate how well a classifier works. With an AUC of 0.9823, the proposed CNN outperformed the baseline models in distinguishing between osteoporosis and normal.

By comparing the suggested 21-layer CNN model with many state-of-the-art deep learning algorithms for osteoporosis diagnosis that use CT, DXA, and X-ray imaging modalities, Table 6 provides a more thorough background and emphasizes current accomplishments. Even while our model’s AUC of 0.9823 was comparable to or better than many other recent methods, it is important to stress that direct comparisons should be handled carefully due to differences in population demographics, dataset size, and imaging modality complexity. Although larger datasets from multi-center cohorts and higher-resolution volumetric information are often advantageous for CT-based investigations, our model uses only two-dimensional DXA spine radiographs, which are scarcer but more readily available in clinical practice. Nonetheless, the suggested CNN demonstrates that lightweight methods for DXA-based osteoporosis screening are feasible because of its similar diagnostic capabilities. To improve generalizability, future research will evaluate our model on datasets from several institutions and compare various imaging modalities.

**Table 6:** Performance comparison of the proposed model with prior studies and research.

Study	Key observations	Reported AUC	Model/architecture	Dataset size/Source	Imaging modality
Proposed model	Lightweight 2D architecture; smaller dataset; competitive AUC despite simpler modality	0.9823	21-Layer CNN	174 DXA images (114 osteoporotic/60 normal)	Spine DXA (2D)
Liu et al. (2025)	External validation; fracture classification; radiomics features integrated	0.96	Deep learning radiomics model	Multi-center, 1,800 patients	Spine CT (vertebral fractures)
Du et al. (2025)	Multi-center dataset; CT radiomics improves bone density classification	0.965	Deep learning + Automatic segmentation + Radiomics	2,500 CT examinations	Proximal femur CT
Liu et al. (2024)	Multi-feature fusion, volumetric CT; transformer module improves feature representation	0.97	Hybrid transformer + CNN radiomics	1,200 patients	Routine CT (vertebral body)
Smith et al. (2024)	Large-scale opportunistic screening, high-resolution 3D CT; near-perfect AUC	~1.00	CNN segmentation + BMD regression	100,000 scans	Chest CT (opportunistic)

## 5 Conclusion

Currently, developed countries are experiencing a rapid increase in osteoporosis cases. Lack of proper treatment and the unavailability of early detection have already led to many lives being lost. In this study, the proposed model uses automated osteoporosis detection from DXA images to aid in the treatment of affected patients. High-level features are extracted from DXA images using a 21-layer CNN model. The osteoporosis dataset contains spine DXA images. The performance achieved through experimentation is significantly better than both the baseline model and state-of-the-art classifiers. We believe our proposed model for automatic osteoporosis detection can assist doctors. In the future, the aim is to address these limitations, as the proposed model has the potential to detect, monitor, and diagnose low BMD, thereby preventing osteoporosis. Advanced approaches can be integrated to further improve research.

## Data Availability

Publicly available datasets were analyzed in this study. This data can be found at: https://data.mendeley.com/datasets/kys6x6wykj/1.
